# Characterization of the spatial distribution of alfalfa weevil, *Hypera postica*, and its natural enemies, using geospatial models

**DOI:** 10.1002/ps.6100

**Published:** 2020-10-13

**Authors:** Govinda Shrestha, Jhalendra P Rijal, Gadi V P Reddy

**Affiliations:** ^1^ Department of Crop and Soil Science, Hermiston Agricultural Research and Extension Center Oregon State University 2121 South 1st street Hermiston OR 97838 USA; ^2^ UC Statewide IPM Program University of California Agriculture and Natural Resources Modesto CA USA; ^3^ USDA‐ARS Southern Insect Management Research Unit Stoneville MS USA

**Keywords:** biological control, geospatial analysis, variogram, SADIE

## Abstract

**BACKGROUND:**

Understanding the spatio‐temporal dynamics of prey and predator distributions can provide valuable insights into pest management strategies and conservation of natural enemies in agro‐ecosystems. The alfalfa weevil, *Hypera postica* (Gyllenhal), is an economically important pest of alfalfa throughout the western United States. Coccinellids and nabids are among the most important natural enemies of this species, contributing to the biological control of *H. postica* in alfalfa fields. The spatio‐temporal dynamics of *H. postica* and these two predator groups were investigated using 81 (= 9 × 9 grid) sample points in each of five alfalfa fields in north‐central Montana. The data were analyzed using variogram and spatial analysis by distance indices (SADIE).

**RESULTS:**

Variogram analysis revealed the spatial dependence (aggregation) of *H. postica* in 17 of 19 sampling times for larvae, and three of 12 sampling times for adults. Using SADIE, statistically significant aggregation distribution was evident in four of 19 sampling times for larvae, and five of 12 sampling times for adults of *H. postica*. Combined variogram and SADIE showed strong evidence of spatial aggregation of *H. postica* larval population (~95%) while a moderate level of aggregation in the adult population (~67%) of the sampling times analyzed. The average aggregation distances based on the range value of the variogram were 22.3 m and 14.7 m for larvae and adults, respectively. Based on variogram results, populations of natural enemies, coccinellids and *Nabis* spp. were found spatially aggregated in 57.9% and 5.6% of the sampling times, respectively. SADIE further supported the variogram results as coccinellid populations (52.6% of sampling times) were highly aggregated in contrast with the *Nabis* spp. populations (5.6% of sampling times) in alfalfa fields. There was no evidence of significant spatial synchrony between *H. postica* and its predators, coccinellids and *Nabis* spp.

**CONCLUSION:**

Our study was able to determine the spatial and temporal distribution of *H. postica* and its two natural enemies (coccinellids and nabids) in irrigated alfalfa fields. The possible implications of these findings for integrated pest management (IPM) of alfalfa weevil populations are discussed.

## Graphical abstract

This study determined the spatial and temporal distribution of *Hypera postica* and the population of its two natural enemies (coccinellids and nabids) in irrigated alfalfa fields.

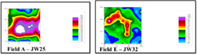

## 1 INTRODUCTION

Alfalfa (*Medicago sativa* L.) is the most important forage plant worldwide.^1^ Alfalfa is considered a superior feed for livestock as it is quickly digestible, high in protein and cell solutes, and a rich source of minerals and vitamins.^2^ In the United States, alfalfa ranks as the fourth most economically important crop, with an estimated annual value of nearly US $8 billion.^3^

The alfalfa weevil, *Hypera postica* (Gyllenhal) (Coleoptera: Curculionidae), is an important specialist herbivore pest of alfalfa. It is believed to be native to Asia, Europe, and North Africa, but has spread to most of the alfalfa‐growing regions of the world.^4^, ^5^ *Hypera postica* has been the most significant pest of alfalfa in the United States since its introduction more than 60 years ago.^4^, ^6^ Both adults and larvae feed on alfalfa, damaging terminals, foliage, and new‐crown shoots, resulting in significant biomass loss, reduced plant growth, and delayed maturity.^7^ Larvae cause most of the damage.^5^, ^8^

In cooler parts of the United States, including Montana, *H. postica* overwinters as an adult. The female becomes active when temperature increases (~ 9 °C threshold) in the spring and lay clusters of 5 to 20 eggs inside stems.^5^, ^9^ After hatching, young larvae move to plant terminals and feed on the folded leaves, while older larvae feed on unfolded plant parts.^10^ Damage from *H. postica* feeding is greatest on the first alfalfa cutting.^11^ The degree of damage to the second and third cuttings depends on the management practices used.^5^, ^7^ The presence of pinholes in alfalfa leaf tissue is an early sign of infestation, while skeletonized leaves indicate heavy weevil damage,^10^, ^12^ which may cause ~50% loss of hay yield in the absence of effective management.^13^, ^14^

As a perennial and nitrogen‐rich plant, alfalfa offers a favorable habitat for many beneficial arthropods, including pollinators and natural enemies.^5^ The presence of effective natural enemies can prevent pest populations from reaching economically damaging levels in alfalfa^15^ and possibly other neighboring crops.^16^, ^17^, ^18^, ^19^, ^20^ Four introduced parasitoid species such as *Bathyplectes curculionis* (Thomson), *B. anurus* (Thomson) (Hymenoptera: Braconidae), *Microctonus aethiopoides* (Loan) (Hymenoptera: Ichneumonidae), and *Oomyzus incertus* (Ratzenberg) (Hymenoptera: Eulophidae) are considered effective natural enemies of alfalfa weevil.^5^ In addition, several species of lady beetles (Coleoptera: Coccinellidae), damsel bugs (Hemiptera: Nabidae)^16^, ^17^, ^18^, ^19^, ^20^ and lacewings (Neuroptera: Chrysopidae)^21^ are known to be major predators of alfalfa weevil. Hence, the presence and abundance of these natural enemies can significantly reduce *H. postica* outbreaks.

Understanding the spatial distribution of a pest and its natural enemies is useful for developing an effective pest monitoring and management program.^22^, ^23^, ^24^, ^25^, ^26^ Insect distribution patterns can be characterized by using mean–variance based methods (Taylor's power law) or spatial methods (spatial analysis by distance indices (SADIE), geostatistics, etc.). The mean–variance methods use the sample mean to variance relationship to characterize the population's distribution as random, aggregated, or over‐dispersed.^27^, ^28^, ^29^ However, these methods lack the explicit ‘spatial distribution of location samples’ in the analysis. The explicit spatial distribution patterns for several arthropod pests have been characterized by using a variety of techniques. For instance, the spatial distribution patterns of tomato leaf miner, *Tuta absoluta* (Meyrick) (Lepidoptera: Gelechiidae), spotted alfalfa aphid, *Therioaphis maculata* (Buckton) (Hemiptera: Aphididae), and corn rootworm, *Diabrotica virgifera* (LeConte) (Coleoptera: Chrysomelidae) were determined in tomato, alfalfa and cornfields, respectively, using a geostatistical analysis.^26^, ^30^, ^31^ This type of analysis can also be used to predict insect distribution patterns during the growing season. A spatial distribution that accounts for the position of the sample points for analysis is desirable over non‐spatial methods. Both spatial and non‐spatial statistics have also been used to determine the distribution patterns of cereal leaf beetle, *Oulema melanopus* (L.) (Coleoptera: Chrysomelidae), in wheat^32^ and grape root borer, *Vitacea polistiformis* (Harris) (Lepidoptera: Sesiidae), in grape vineyards.^33^ Knowledge of a pest's spatial distribution can be useful in the development of sampling plans by identifying the minimum inter‐sample distance needed to obtain independent samples,^26^, ^34^, ^35^ improving insecticide‐resistance management, and conserving beneficial insects.^36^, ^37^, ^38^ Understanding of the insect spatial distribution is critical for site‐specific pest management.

The spatial distribution of natural enemies helps to understand the pest–natural enemy relationship in the field.^22^, ^25^, ^34^, ^39^ Natural enemies (e.g. predators and parasitoids) can disperse to find the patches of high pest densities in the field.^40^, ^41^ Understanding the spatio‐temporal dynamics of pests and their natural enemies within a field is also important for the conservation and release of biological agents in the field,^24^ as the success of biological control is higher when there is a spatio‐temporal overlap of prey and natural enemies.^24^, ^38^ Despite the usefulness of spatio‐temporal distribution tools to understand the ecology of pests and their natural enemies, a limited number of studies have been conducted to determine the spatio‐temporal associations of pests and their key natural enemies.^23^, ^24^, ^26^, ^39^

The spatial distribution and association of pests and their natural enemies can be characterized using several methods, including variogram and SADIE. Both methods have their strengths and weaknesses, and the combination of the two methods is recommended in ecological studies.^33^, ^42^, ^43^ In alfalfa fields, two important natural enemy guilds, coccinellids, and nabids prey on alfalfa weevils.^18^, ^19^, ^20^ Although these two predator groups play an important ecological role in balancing the prey–natural enemy dynamics in alfalfa fields, no information is available on the spatio‐temporal distribution of *H. postica* and these natural enemies. Therefore, this study aimed to determine the spatio‐temporal distribution of *H. postica*, and two natural enemy groups – coccinellids, and nabids using two geospatial methods, SADIE and variogram.

## 2 MATERIALS AND METHODS

### 2.1 Study sites and crop production practice

Five irrigated commercial alfalfa fields were selected from three locations: Conrad (Field A: N 48^o^ 35.192 W112^o^ 21.169; and Field B: N 48^o^ 30.206 W112 ^o^14.350), Ledger (Field C: N 48^o^ 35.192 W112^o^ 21.169; and Field D: N 48^o^ 35.192 W112^o^ 21.169) and Valier (Field E: N 48^o^ 35.192 W112^o^ 21.169), in Pondera County, Montana, USA. All fields were in north‐central Montana. Alfalfa plants were grown multiple years on the same piece of land after broadcast seeding with 1–2 harvesting (i.e. cuttings) per year; therefore, plant stand age in the fields used in this study ranged from two to five years. Field sizes (A, B, C, D and E) were 40, 16, 30, 12 and 68 ha, respectively. Irrigation was applied one to two times before and after alfalfa cutting using wheel‐line or center‐pivot systems. None of the fields received insecticides during the growing period of the sampling year nor in the two previous years (2014–2015). Two cuttings were made, which is the typical harvest practice for irrigated alfalfa in Montana, where the active growing season for alfalfa is between May and August. The average seasonal temperature (May–August) for Conrad, Ledger and Valier fields ranged 10–18, 9–18 and 9–17 °C, respectively.^44^

### 2.2 Sampling

Samplings of *H. postica* (larvae and adults), lady beetle larvae and adults (Coccinellidae), and damsel bugs nymphs and adults (Nabidae) were conducted in a portion (i.e. sampling area ~0.2 ha) of the five fields in 2016. The sampling area contained 81 sampling points distributed at 5 m intervals in a square grid (i.e. nine sampling points across *X*‐coordinates and nine sampling points across *Y*‐coordinates) marked with 1‐m tall red‐painted wooden sticks. Sampling was conducted using a standard 180°‐sweep net (diameter 38 cm) sampling (ipm.ucanr.edu/PMG/r1300511.html), taking 20 sweeps at each sampling point (distributed as five sweeps in each cardinal direction from each sample point). The collected insect samples were placed in plastic Ziploc® bags and taken to the laboratory on the day of collection, where they were either processed immediately or frozen at −20 °C for later identification and counting. Samples were collected on four dates: two before and two after the first alfalfa cutting. After the second sampling (before first cutting), wooden sticks were removed from study sites and replaced with plastic ear tags. Before the start of the third sampling, plastic ear tags were removed, and wooden sticks were relocated to the study sites. The placement of plastic ear tags allowed us to find the same spots that were sampled before the first alfalfa cutting. Sampling was performed roughly every 10‐days: first sampling from June 1–3 [i.e. Julian week (JW) 23]; second sampling from June 14–16 (i.e. JW 25); third sampling from July 27–29 (i.e. JW 31); and fourth sampling from August 5–7 (i.e. JW 32). Sampling was conducted 3–4 times for each field with a total of 19 samplings across five fields, and these sampling instances were hereafter referred to as sampling times.

### 2.3 Geospatial analysis

Two different geospatial techniques, that is the variogram and SADIE, were used to characterize the spatial distribution of *H. postica* and its two natural enemies in alfalfa fields. SADIE accounts for the underlying patchiness of insect counts from spatially referenced locations, whereas the variogram assumes a gradual change in abundance for local insect populations, thus both methods can produce significantly different results and conclusions.^45^, ^46^ In order to avoid potential bias from data analysis techniques, datasets from the specific sampling dates that yielded a minimum total of ten insect counts from the entire sampling area were subjected to variogram and SADIE analyses. Using this criterion, data from the total 19, 12, 19, and 18 sampling times for *H. postica* larva, *H. postica* adult, coccinellids, and *Nabis* spp. respectively, were used for geospatial analyses, namely variogram and SADIE.

### 2.4 Variogram

Variogram plots depict spatial dependence by calculating the autocorrelation among sample points, making them a geostatistical method for determining the spatial distribution pattern of arthropods.^47^, ^48^ Spatial dependence is determined by developing an experimental variogram that describes the relationship between sample values and distance and direction within the sampling space. Mathematically, the variogram (*γ*) is calculated as follows:^47^, ^48^

$$\hat{\gamma }\left(h\right)=\frac{1}{2N\left(h\right)}\;\sum_{i=1}^{N\left(h\right)}{\left\lfloor z\left(x_{i}\right)-z\left(x_{i}+h\right)\right\rfloor }^{2},$$

where $\hat{\gamma }\left(h\right)$ is the estimated semivariance for the entity of interest (*z*) at all points (*x*_*i*_) separated by lag distance (*h*), and *N*(*h*) is the number of pairs of samples separated by lag distance *h*.

Insect counts that did not meet the assumption of normality were transformed using log(*x* + 1). We used either direct insect count data or the transformed data for variogram analysis. For variogram model development, it is critical to remove large‐scale variation (trend) that may exist in the data.^47^, ^49^ Multiple linear regression analysis was used to determine the trend for data for individual sampling dates by using insect counts as the dependent variable and the spatial references (i.e. *X* and *Y* values) of individual sample points as the independent variables.^30^ A significant regression (*P* < 0.05) indicated the presence of the trend in the dataset, and the standard residuals of those datasets were used to develop variograms. Out of 19 sampling weeks, standard residuals were used in six, four, four, and ten datasets of *H. postica* adult, *H. postica* larvae, *Nabis* spp., and coccinellids respectively to develop variograms. Variograms were developed using geostatistical software, GS^+^ (version 9.0.11).^50^

Three parameters (i.e. range, sill, and nugget) of the variogram model determine the shape of the plots. The maximum distance over which the spatial dependence persists is called the range,^51^, ^52^ the semivariance value at which the variogram plot plateaus is the sill, while the semivariance value at zero lag distance is termed the nugget.^51^ Straight‐line plots (i.e. nugget or linear variogram models) do not have a definite sill are indicative of the random distribution pattern.^53^, ^54^ The curvilinear plots (i.e. exponential, spherical, or Gaussian) have a definite sill, which indicates the existence of spatial dependence (i.e. aggregation).^49^, ^55^, ^56^ We used omnidirectional variograms,^49^, ^51^ which produce more accurate and discernible results than any other directional types of variogram^49^ and have been used in previous studies.^33^, ^53^, ^57^ The best fitted omnidirectional variograms were selected based on the greatest *r*^2^ value.^30^, ^33^, ^35^, ^58^ Nugget‐to‐sill ratios (*C*_0_/*C*_0_ + *C*) describe the extent of aggregation,^59^ where ratios of < 0.25, 0.25–0.75, and > 0.75 indicate strong, moderate, and weak aggregation, respectively.^33^, ^35^, ^43^, ^58^, ^60^

### 2.5 Spatial analysis by distance indices (SADIE)

SADIE is a geospatial technique that can be used to determine the spatial distribution pattern of arthropod pests and plant diseases using spatially referenced count data.^33^, ^42^, ^43^ SADIE measures the overall aggregation based on the distance to regularity (*D*), which represents the minimum total distance that individual samples would need to move in order to obtain the same number (i.e. mean) for individual sample points. The aggregation is expressed in the form of clustering areas with either greater (i.e. patches) or smaller counts (i.e. gaps) compared to the mean. The magnitude of *D* can be calculated by a randomization test in which permutations of all observed counts from sample points are performed.^45^ The assessment provides an index of aggregation, *I*_a,_ and probability, *P*_a_. Values, *I*_a_ > 1, *I*_a_ = 1, and *I*_a_ < 1, indicate the aggregation, random, and uniform distribution patterns. The associated probability (i.e. *P*_a_ < 0.025) determines the statistical significance of the resultant distribution pattern.^33^, ^42^, ^61^ The SADIE analysis was carried out using SADIE Shell (version 2).^61^ In total, 153 permutations and 10 000 randomizations with a non‐parametric option were used for SADIE analysis.

### 2.6 Spatial association of *H. postica* with its natural enemies

The spatial association between two datasets was conducted using N_AShell (version 1.0), a part of the SADIE.^61^ Spatial association, indicated by the index of spatial association (*X*_0_) was used to determine any spatial synchrony between *H. postica* and two natural enemies, *Coccinellid* spp., and *Nabis* spp. This information may better explain the ecological roles of different factors in spatial distribution and sampling. Significant positive association (*X* > 0; *P* < 0.025) indicates the presence of either a gap or a patch for both variables (i.e. *H. postica* and the natural enemy population) in that particular week of sampling while significant negative association (*X* < 0; *P* < 0.975) indicates association of a patch of one variable with a gap of the other variable or vice versa.^62^

## 3 RESULTS

### 3.1 Temporal distribution of *H. postica* infestation

**Figure 1:**
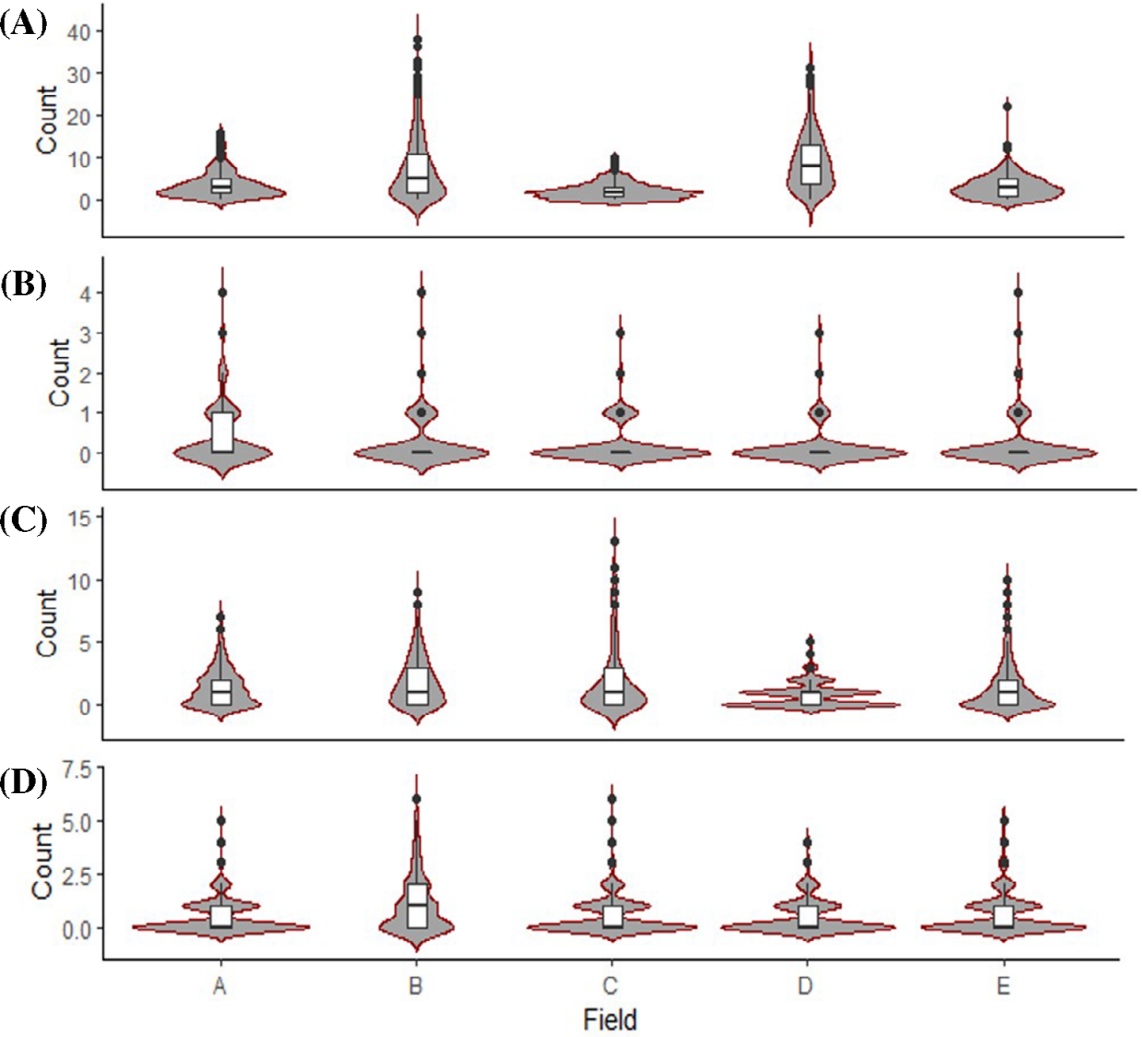
Violin plots showing the distribution of total numbers of insect counts per 20 sweeps from sampling area of five alfalfa fields in north‐central, Montana: (A) *Hypera postica* larvae, (B) *H. postica* adults, (C) coccinellids and (D) *Nabis* spp. The data were pooled from three to four sampling dates at each field.

**Figure 2:**
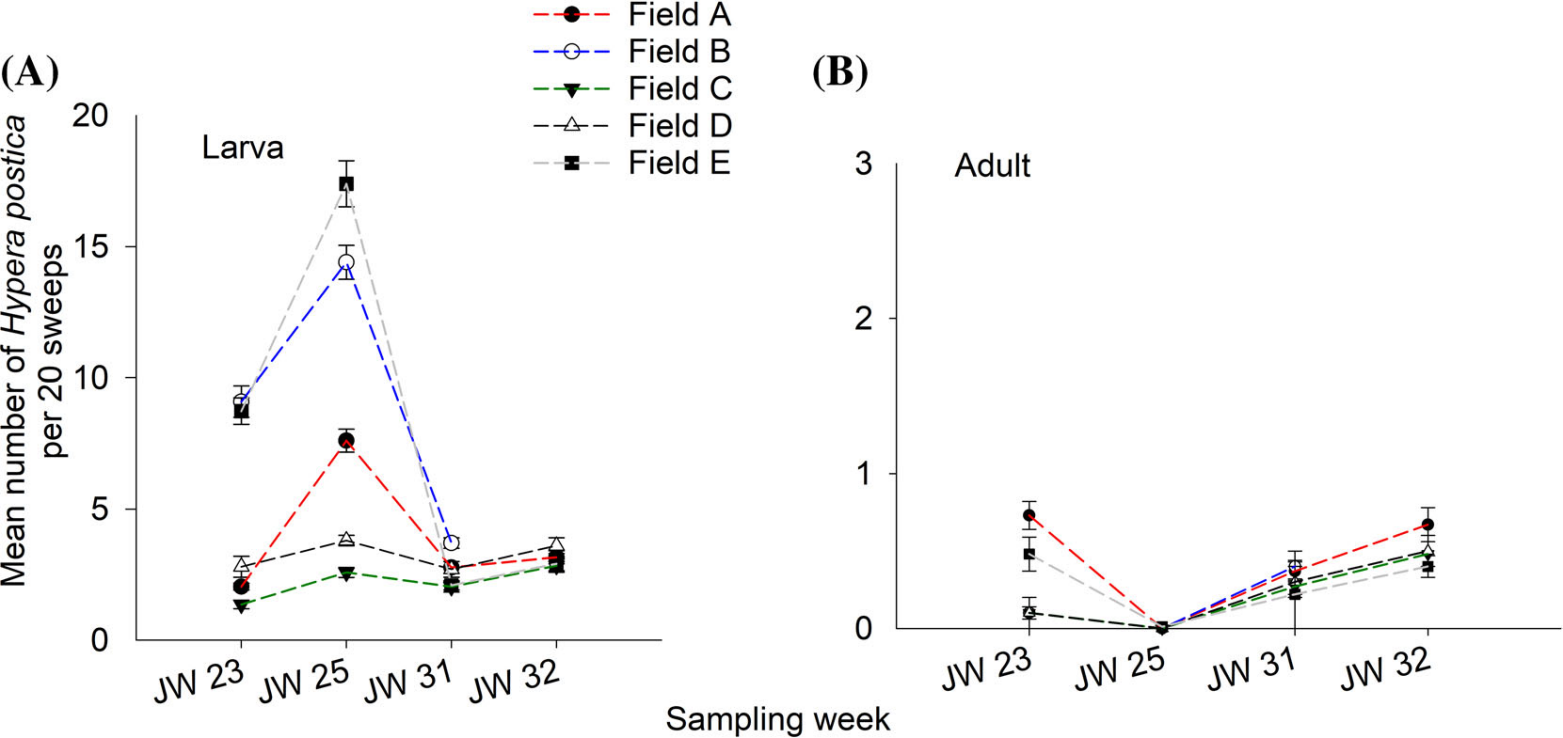
Mean (± standard error) number of *Hypera postica* (A) larvae and (B) adults in five alfalfa fields in north‐central, Montana. Means were calculated based on the total number of insect individuals counted per 20 sweeps. JW, Julian week.

A total of 7474 *H. postica* larvae was collected across all five fields. The overall distribution of larvae at each field was presented in violin plot (Fig. 1(aA)). The degree of *H. postica* larval infestation varied based on the sampling date and field (Fig. 2(A)). At the first sampling date (JW 23), there was nearly four‐fold higher larvae mean infestations levels in Fields B and E (nine larvae per 20 sweeps) compared to the Fields A, C and D (1–2 larvae per 20 sweeps) (Fig. 2(A)). In the second sampling date, (JW 25), there was a 10–50% increase in mean larval numbers across five fields (Fig. 2(A)). In contrast, after first alfalfa cutting [i.e. third (JW 31) and fourth (JW 32) samplings], mean larvae infestation levels sharply declined in all fields, regardless of sampling date (Fig. 2(A)). For *H. postica* adults, densities were low in all five alfalfa fields, with a mean population level of < 1 adult per 20 sweeps (Fig. 2(B)). A total of 418 adults was collected across all five fields and the total distribution of adults at each field was presented in violin plot (Fig. 1(B)). Adults were observed mainly either in JW 23 or in the last two sampling weeks (JWs 31 and 32). No adult activity was observed in JW 25 across all five fields (Fig. 2(B)).

### 3.2 Temporal distribution of natural enemies of *H. postica*

**Figure 3:**
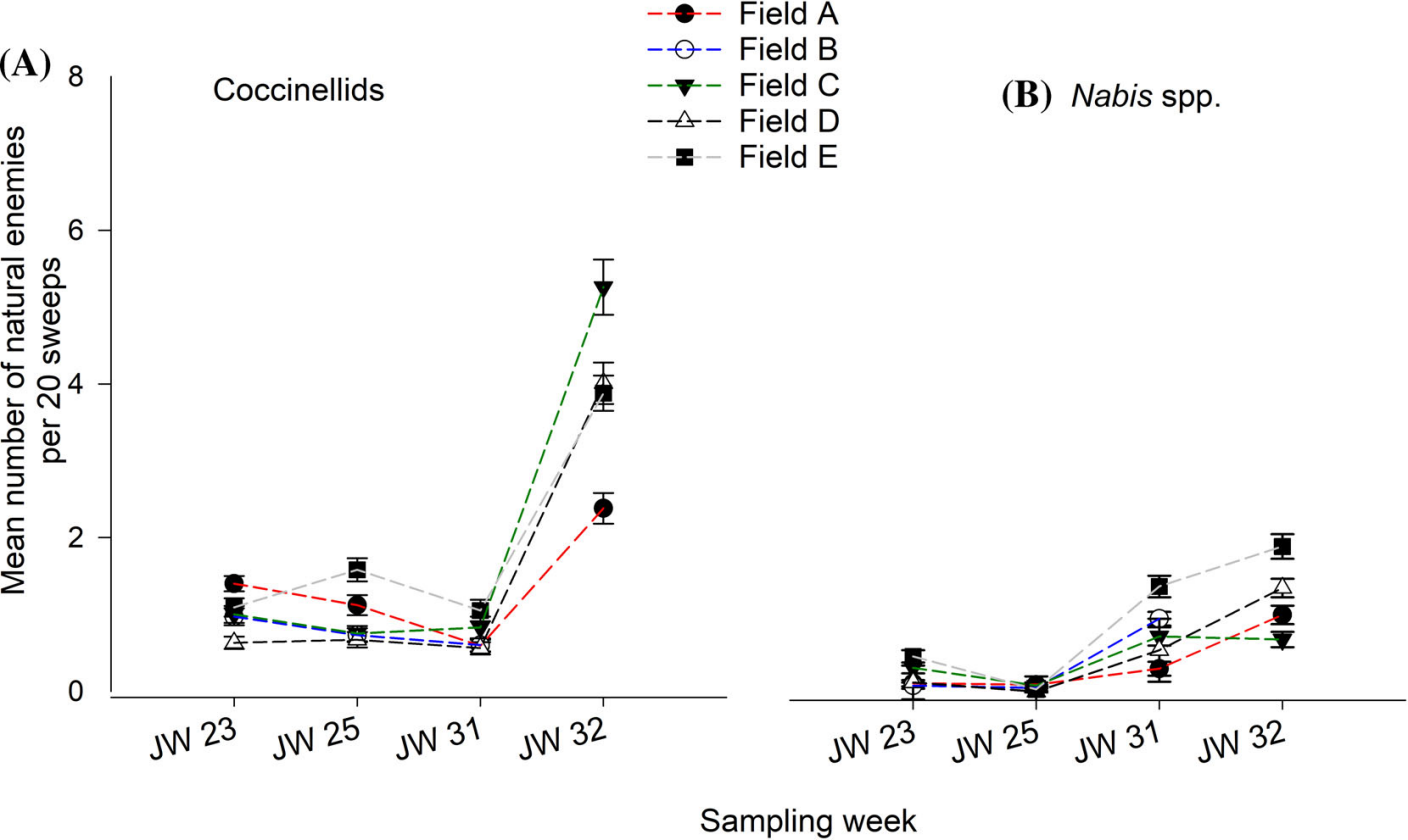
Mean (± standard error) number of *Hypera postica* predators (A) coccinellids and (B) *Nabis* spp., in five alfalfa fields in north‐central, Montana. Means were calculated based on the total number of insect individuals counted per 20 sweeps. JW, Julian week.

The two *H. postica* predator groups (Coccinellidae and Nabidae) were found in all five fields. The total numbers of coccinellids and nabids collected were 2356 and 988, respectively across five fields. The overall distribution of predators at each field was presented in violin plot (Fig. 1(C, D)). The coccinellid composition was dominated by the introduced seven‐spotted lady beetle (*Coccinella septempunctata* L.) (> 97% of the collected samples), followed by the two‐spotted lady beetle (*Adalia bipunctata* L.) (Coleoptera: Coccinellidae) across five fields. Coccinellid population density fluctuated depending on sampling time and field location. At JW 23, the mean coccinellid population density was 1.6 times higher in Fields A, B, C and E contrasted to Field D (Fig. 3(A)). At JWs 25 and 31, the population density generally remained constant across all fields, except for Field A in which the population‐level was reduced by half compared to JW 23 sampling date (Fig. 3(A)). However, coccinellid populations increased in all five fields at the final sampling date (JW 32), with 4–5 larvae or adults per 20 sweeps in Fields C, D and E compared to Field A (two larvae or adults per 20 sweeps) (Fig. 3(A)).

In comparison to coccinellids, nabids were less abundant in all alfalfa fields sampled. Nabid composition was largely dominated by *Nabis americoferus* Carayon and *N. ferus* L., (Hemiptera: Nabidae) in similar proportions in all locations. Among the five field locations, Field C generally had a lower population density throughout the sampling times, except the minimal level of increase at JWs 31 and 32. The mean level varied from 0.19 to 0.83 per 20 sweeps. In Fields A and D, nabid density remained relatively unchanged for earlier sampling dates (i.e. JWs 23 and 25) until JW 31, but increased two‐fold by the last sampling time, JW 32. In Field E, nabid. density increased sharply after the first alfalfa cutting (i.e. JWs 31 and 32) with mean densities of 1.50 and 2.00 adults per 20 sweeps at JWs 31 and 32, respectively (Fig. 3(B)).

### 3.3 Within‐field distribution of *H. postica*

#### 3.3.1 *Spatial aggregation of* **H. postica** *using variograms*

**Figure 4:**
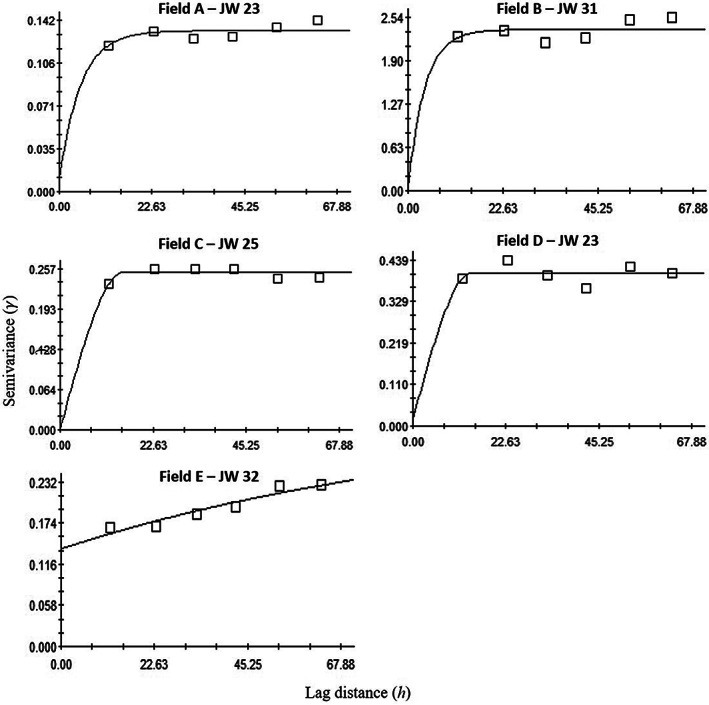
Examples of variograms showing the spatial distribution of *Hypera postica* larvae in five alfalfa fields (Fields A–E). JW, Julian week.

Among five fields, variograms indicated the aggregated distribution pattern of *H. postica* larvae in 17 of 19 sampling times that included all sampling times for three fields: Field A (JWs 23, 25, 31 and 32), Field B (JWs 23, 25 and 31) and Field D (JWs 23, 25, 31 and 32) (Table 1; Fig. 4). In other field locations, it was found in three sampling times of Field C (JWs 23, 25 and 32) and Field E (JWs 23, 25 and 31) (Table 1).

**Table 1:** Best fitted variogram models and parameters representing the spatial distribution of *Hypera postica* larva and adult in alfalfa fields

Field	Sampling week	Larva				Adult			
		range (m)	Model	*r* ^2^	*C*_0_/*C*_0_ + *C*	range (m)	Model	*r* ^2^	*C*_0_/*C*_0_ + *C*
A	JW 23	15.60	Ex	0.474	0.077	17.30	Ex	0.370	0.090
	JW 25	53.20	Sp	0.838	0.359	—	—	—	—
	JW 31	27.6	Ex	0.621	0.111		Li	0.09	—
	JW 32	12.47	Ga	0.178	0.073	—	Li	0.795	—
B	JW 23	14.40	Sp	0.058	0.040	—	—	—	—
	JW 25	23.10	Ex	0.742	0.121	—	—	—	—
	JW 31	13.16	Ga	0.381	0.062	—	Li	0.696	—
C	JW 23	17.70	Ex	0.400	0.091	—	—	—	—
	JW 25	15.50	Sp	0.508	0.001	—	—	—	—
	JW 31	—	Li	0.812	—	—	Li	0.458	—
	JW 32	15.00	Ex	0.500	0.087	—	Li	0.05	0.950
D	JW 23	18.00	Ex	0.408	0.001	—	Li	0.295	—
	JW 25	21.60	Ex	0.922	0.419	—	—	—	—
	JW 31	54.70	Sp	0.884	0.329	12.80	Sp	0.02	0.034
	JW 32	8.70	Ex	0.03	0.084	—	Li	0.07	0.959
E	JW 23	21.00	Ex	0.24	0.001	—	Li	0.454	—
	JW 25	8.10	Ex	0.02	0.080	—	—	—	—
	JW 31	39.50	Sp	0.67	0.011	14.10	Sp	0.052	0.001
	JW 32	—	Li	0.798	—	—	Li	0.38	—

Note: *C*_0_, nugget; *C*_0_ + *C*, sill; *C*_0_/*C*_0_ + *C*, nugget‐to‐sill ratio; Nu, nugget model (*C*_0_ = *C*_0_ + *C*); Ga, Gaussian model; Ex, exponential model; Sp, spherical model; Li, linear model; JW, Julian week.

Missing cells for the range and *C*_0_ */C*_0_ *+ C* categories indicate that the selected models do not have those outcomes. Missing cells for the model and *r* ^2^ categories indicate that data was insufficient for conducting variogram analysis. The total numbers of larvae and adults collected were 7474 and 418 respectively across five fields.

Data were primarily fitted to an exponential variogram model (*n* = 10) followed by spherical (*n* = 5), Gaussian (*n* = 2) and linear (*n* = 2). The nugget‐to‐sill ratios which measure the degree of aggregation of *H. postica* larvae were < 0.25 (i.e. strong aggregation), 0.25–0.75 (i.e. moderate aggregation), and > 0.75 (i.e. weak aggregation) in 14, three, and zero of 17 sampling times, respectively (Table 1).

**Figure 5:**
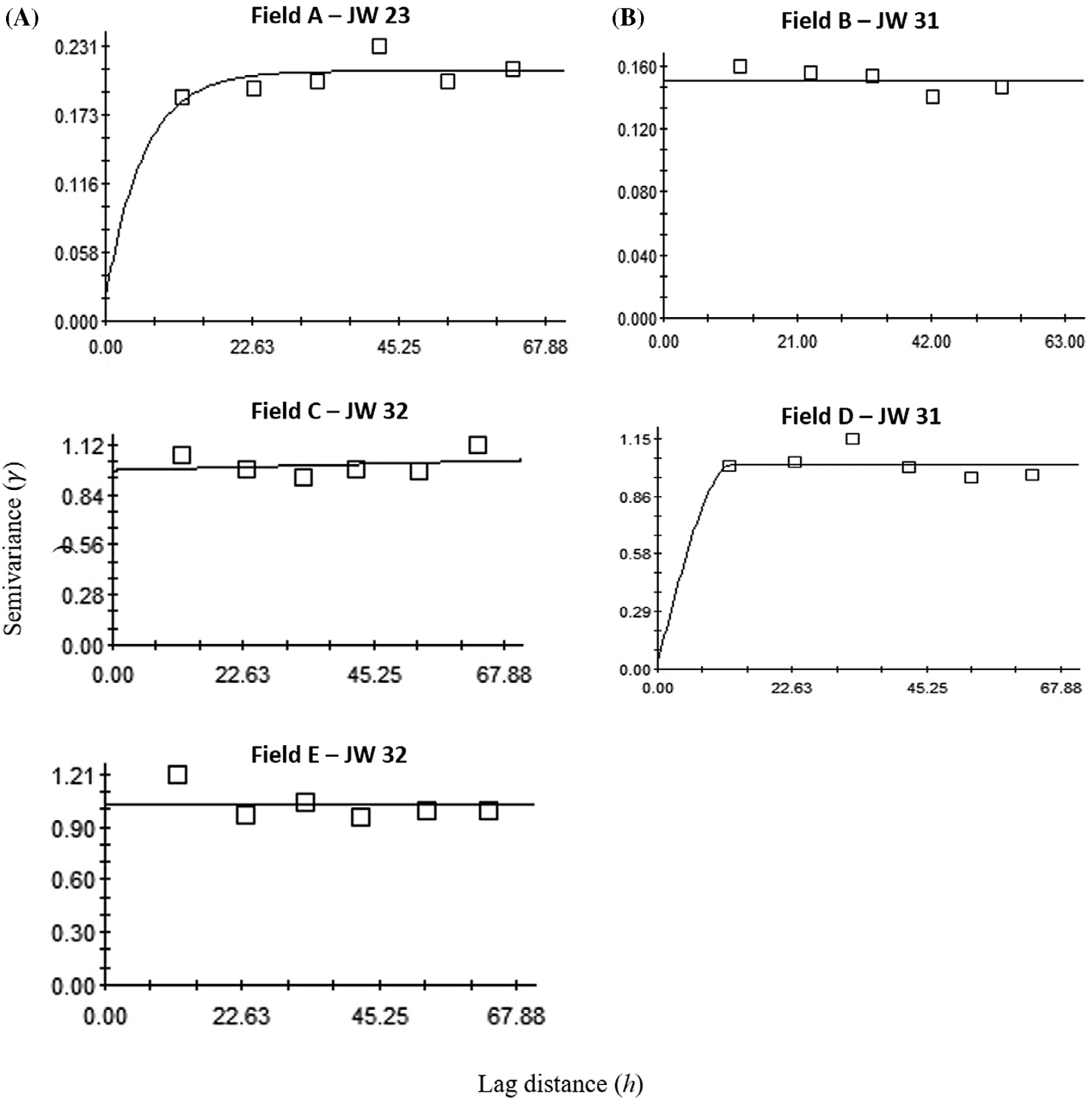
Examples of variograms showing the spatial distribution of *Hypera postica* adults in five alfalfa fields (Fields A–E). JW, Julian week.

Contrary to larval population, *H. postica* adult aggregation distribution patterns were found in only three of 12 sampling times: JW 23 for Field A; JW 31 for Field D; and JW 31 for Field E (Table 1; Fig. 5). Data were mainly fitted to a linear variogram model (*n* = 9) followed by spherical (*n* = 2) and exponential (*n* = 1). All three sample times showed a strong spatially aggregated distribution of adults, as indicated by < 0.25 nugget‐to‐sill ratio (Table 1). The range value of variogram represents the distance of aggregation. Range values of the variograms of *H. postica* larvae were between 8.1 m and 54.7 m, with a mean of 22.3 m, while range values of the variograms of *H. postica* adults were between 12.3 and 17.3 with a mean of 14.7 m (Table 1).

#### 3.3.2 *Spatial aggregation of* **H. postica** *using* *SADIE*

Using SADIE, the aggregated distribution pattern for *H. postica* larvae was statistically significant (*P* < 0.025) in four of 19 sampling times. Regarding adult distribution, five of 12 sampling times showed a statistically significant aggregation pattern (*P* < 0.025) (Table 2).

**Table 2:** Spatial analysis by distance indices (SADIE) parameters for *Hypera postica* larva and adult distribution in alfalfa fields

Field	Sampling week	Larvae		Adults	
		*I*_a_	*P*_a_	*I*_a_	*P*_a_
A	JW 23	1.141	0.158	1.069	0.269
	JW 25	1.484	0.0084*	—	—
	JW 31	1.120	0.191	1.899	<0.001**
	JW 32	1.131	0.182	2.138	<0.001**
B	JW 23	1.192	0.111	—	—
	JW 25	1.265	0.061	—	—
	JW 31	0.875	0.818	0.875	0.818
C	JW 23	1.183	0.110	—	—
	JW 25	1.036	0.337	—	—
	JW 31	1.074	0.264	0.740	1.000
	JW 32	1.099	0.222	2.048	<0.001**
D	JW 23	1.153	0.144	0.981	0.477
	JW 25	1.094	0.291	—	—
	JW 31	1.646	0.0018**	0.877	0.807
	JW 32	1.698	0.001**	1.407	0.015*
E	JW 23	0.854	0.854	1.149	0.147
	JW 25	1.094	0.291	—	—
	JW 31	0.740	0.894	1.209	0.091
	JW 32	1.405	0.0178*	1.398	0.017*

Note: *I*_a_, index of aggregation; *P*_a_, *P* value of *I*_a_; JW, Julian week. Missing cells indicates that insect counts were insufficient to conduct aggregation analysis.

* Significant at *P* < 0.025.

** Significant at *P* < 0.005.

### 3.4 Within‐field distribution of *H. postica* natural enemies

#### 3.4.1 *Spatial aggregation of natural enemies of* **H. postica** *using variograms*

**Figure 6:**
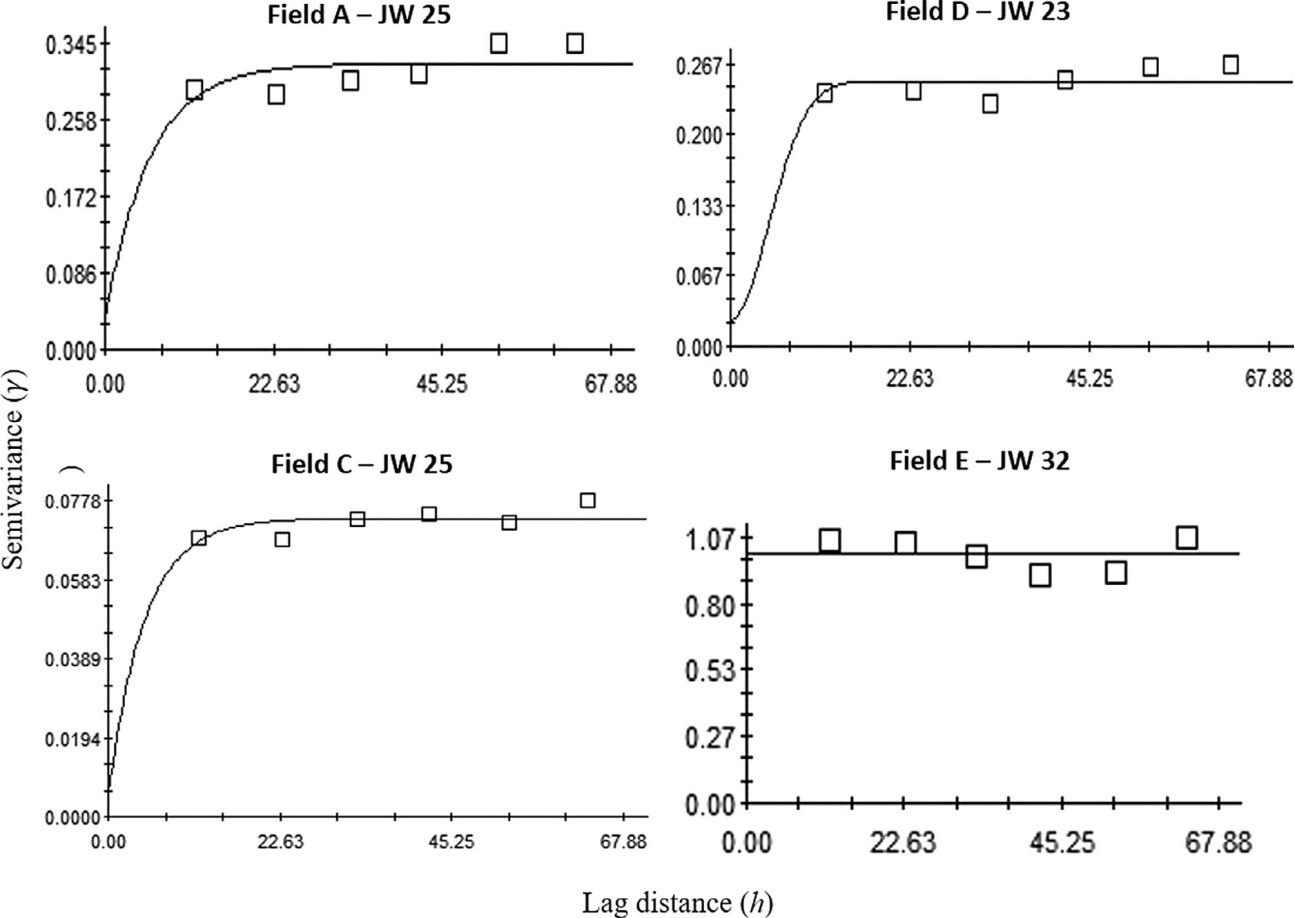
Examples of variograms showing the spatial distribution of coccinellids (top two graphs), and *Nabis* spp. (bottom two graphs) in alfalfa fields. JW, Julian week.

Spatial aggregations were detected in 12 of 19 sampling dates for coccinellids, but only in one sampling time for nabids (Table 3). In JW 23, coccinellid populations were aggregated in all fields, and in three fields (Fields A, C and E; Table 3) in JW 32. In contrast, coccinellid populations were aggregated in only two fields (Fields A and D) at JW 25, and two fields (Fields B and C) at JW 31. Regarding the nabid population, aggregation pattern was observed in one sampling time (i.e. JW 31 of Field C) (Table 3; Fig. 6).

**Table 3:** Best fitted variogram models and parameters representing the spatial distribution of coccinellids and *Nabis* spp. in alfalfa fields

Field	Sampling week	Coccinellids				*Nabis* spp.			
		range (m)	Model	*r* ^2^	*C*_0_ */C*_0_ *+ C*	Range (m)	Model	*r* ^2^	*C*_0_ */C*_0_ *+ C*
A	JW 23	—	Li	0.28	—	—	Li	0.218	—
	JW 25	18.00	Ex	0.259	0.101	—	Li	0.228	—
	JW 31	—	Li	0.150	—	—	Li	0.100	—
	JW 32	13.60	Sp	0.07	0.011	—	Li	0.423	—
B	JW 23	13.20	Ex	0.94	0.001	—	Li	0.251	—
	JW 25	—	Li	0.000	—	—	Li	0.066	—
	JW 31	13.85	Ga	0.906	0.134	—	Li	0.000	—
C	JW 23	13.10	Sp	0.002	0.023	—	Li	0.215	—
	JW 25		Li	0.33	—	—	Li	0.33	—
	JW 31	16.60	Sp	0.677	0.034	13.20	Ex	0.10	0.041
	JW 32	25.50	Ex	0.73	1.083	—	Li	0.75	—
D	JW 23	15.90	Ex	0.300	0.065	—	Li	0.728	—
	JW 25	21.60	Ex	0.247	0.055	—	—	—	—
	JW 31	—	Li	0.03	—	—	Li	0.187	—
	JW 32	20.40	Li	0.38	—	—	Li	0.010	—
E	JW 23	13.90	Sp	0.06	0.10	—	Li	0.069	—
	JW 25	—	Li	0.68	—	—	Li	0.62	—
	JW 31	—	Li	0.337	—	—	Li	0.439	—
	JW 32	13.16	Ga	0.713	0.043	—	Li	0.09	—

Note: *C*_0_, nugget; *C*_0_ + *C*, sill; *C*_0_/*C*_0_ + *C*, nugget‐to‐sill ratio; Nu, nugget model (*C*_0_ = *C*_0_ + *C*); Ga, Gaussian model; Ex, exponential model; Sp, spherical model; Li, linear model; JW, Julian week.

Missing cells for the range and *C*_0_ */C*_0_ *+ C* categories indicate that the selected models do not have those outcomes. Missing cells for the model and *r* ^2^ categories indicate that data was insufficient for conducting variogram analysis. The total numbers of coccinellids and nabids collected were 2356 and 988 respectively across five fields.

When data were fitted to the variogram models, most coccinellid data fitted the linear (*n* = 8) compared to the exponential (*n* = 5), spherical (*n* = 4), or Gaussian (*n* = 2). Conversely, 17 of 18 nabid data fitted the linear (*n* = 17) model, while one dataset (i.e. JW 31 of Field C) fitted to the exponential model. The nugget‐to‐sill ratio was < 0.25, indicating strong aggregation in 11 and two of the sampling times for coccinellids and nabids, respectively (Table 3). Besides, moderate aggregation (nugget‐to‐sill ratio, 0.25–0.75) was detected in one sampling time for coccinellid.

The range value of the variogram represents the distance of spatial dependency (i.e. aggregation). The range values of the variograms of coccinellids were between 13.1 m and 25.6 m, with a mean of 16.6 m, while only one sampling date showed aggregation in *Nabis* spp. with a range value of 13.2 m (Table 3).

#### 3.4.2 *Spatial aggregation of natural enemies of* **H. postica** *using* *SADIE*

The coccinellid aggregation was statistically significant (*P* < 0.025) in 10 of 19 sampling times, with at least one significant sampling date occurring in each field (Table 4). In contrast, aggregation of nabids was only significant in one of 18 sampling times, as was evident in Field C (Table 4).

**Table 4:** Spatial analysis by distance indices (SADIE) parameters for coccinellids and *Nabis* spp. distribution in alfalfa fields

Field	Sampling week	Coccinellids		*Nabis* spp.	
		*I*_a_	*P*_a_	*I*_a_	*P*_a_
A	JW 23	1.603	0.0027**	0.910	0.694
	JW 25	1.357	0.0261	1.032	0.345
	JW 31	0.920	0.669	1.018	0.366
	JW 32	1.544	0.0049**	0.968	0.508
B	JW 23	1.440	0.0127*	1.157	0.145
	JW 25	0.912	0.681	1.296	0.051
	JW 31	0.920	0.661	0.955	0.542
C	JW 23	1.090	0.235	1.159	0.127
	JW 25	1.269	0.056	1.407	0.0169*
	JW 31	2.050	0.0002**	1.075	0.247
	JW 32	1.551	0.003**	1.306	0.0412
D	JW 23	1.386	0.0171*	0.929	0.6325
	JW 25	2.006	0.0002**	—	—
	JW 31	0.790	0.790	0.828	0.9375
	JW 32	1.199	0.104	1.332	0.0315
E	JW 23	1.458	0.0151*	0.871	0.8277
	JW 25	0.851	0.865	1.049	0.310
	JW 31	1.401	0.0178*	0.806	0.952
	JW 32	1.652	0.0013**	0.978	0.4857

Note: *I*_a_, index of aggregation; *P*_a_, *P* value of *I*_a_; JW, Julian week. 1

* Significant at *P* < 0.025.

** Significant at *P* < 0.005.

### 3.5 Spatial association of *H. postica* with its natural enemies

There was not a significant positive association of the *H. postica* population with its two predator groups, coccinellid and nabid across all sampling times across five fields. In Field A, there was a significant negative association between *H. postica* larvae and coccinellids in only one of 19 sampling dates (Table 5).

**Table 5:** Spatial analysis by distance indices (SADIE) spatial association parameters for *Hypera postica* and its natural enemies, coccinellids and *Nabis* spp. in alfalfa fields

Field	Sampling week	*Hypera postica* larvae *versus* coccinellids		*Hypera postica* adults *versus* coccinellids		*Hypera postica* larvae *versus Nabis* spp.		*Hypera postica* adults *versus Nabis* spp.	
		*X*_a_	*P*	*X*_a_	*P*	*X*_a_	*P*	*X*_a_	*P*
A	JW 23	−0.238	0.977*	0.083	0.244	−0.039	0.669	−0.037	0.556
	JW 25	0.041	0.350	—	—	0.128	0.128	—	—
	JW 31	0.217	0.039	0.036	0.415	0.076	0.264	0.156	0.116
	JW 32	0.090	0.225	0.122	0.154	0.102	0.188	0.023	0.429
B	JW 23	−0.016	0.536	−0.021	0.478	−0.080	0.747	−0.075	0.563
	JW 25	−0.186	0.944	—	—	−0.004	0.485	—	—
	JW 31	0.012	0.469	0.006	0.501	0.011	0.470	0.001	0.509
C	JW 23	−0.130	0.849	—	—	−0.087	0.755	—	—
	JW 25	−0.125	0.870	—	—	0.042	0.386	—	—
	JW 31	0.030	0.390	−0.046	0.600	0.179	0.079	0.159	0.101
	JW 32	0.217	0.027	0.202	0.033	0.112	0.162	0.195	0.076
D	JW 23	0.002	0.491	—	—	−0.050	0.666	—	—
	JW 25	0.210	0.037	—	—	0.060	0.283	—	—
	JW 31	0.143	0.122	0.023	0.445	−0.039	0.616	−0.036	0.581
	JW 32	0.016	0.445	0.245	0.017	−0.035	0.617	0.079	0.249
E	JW 23	−0.218	0.983	0.056	0.357	−0.041	0.585	−0.046	0.723
	JW 25	0.156	0.210	—	—	−0.040	0.113	—	—
	JW 31	0.047	0.337	0.068	0.224	0.069	0.272	0.230	0.032
	JW 32	0.159	0.089	0.074	0.262	0.047	0.322	0.143	0.087

Note: *X*_a_, index of association; JW, Julian week. Missing cells indicates that insect counts were insufficient to conduct association analysis.

^*^ Significant at *P* < 0.025 (positive association) or at 0.975 (negative association).

## 4 DISCUSSION

Our study characterized the spatio‐temporal relationship of a major alfalfa pest, *H. postica*, and two groups of its predators in irrigated alfalfa fields in Montana using variogram and SADIE. These two methods, when used for the dataset, can produce different results due to the different ways to calculate the spatial weights for individual sample points.^46^, ^63^ Since SADIE measures clustering among neighboring sample points, some isolated higher values of individual sample points do not contribute to aggregation. In contrast, variogram analysis incorporates these higher values in characterizing the local population distribution.^45^, ^46^ Also, in some instances, the asymptotic models of the variogram do not fit adequately with the experimental data due to small *r*^2^ values. Therefore, combining two methods is recommended to address the discrepancy between these two methods,^46^ and have been used in several previous studies.^35^, ^46^, ^63^, ^64^

By combining variogram and SADIE results, the spatial aggregation of *H. postica* infestations was detected in all five study locations. Spatial aggregations of *H. postica* larvae and adults were detected in ~95% and ~ 67%, respectively, of all sampling times. These results suggested the strong spatial and temporal aggregation of *H. postica* larvae, while moderate aggregation was observed for adults in these irrigated alfalfa fields.

Alfalfa weevil aggregation has been reported as the most typical distribution pattern in a variety of agro‐ecosystems using mean–variance based methods.^65^, ^66^, ^67^ Using Taylor's power law and Iwao's index, Latheef and Pass^65^ and Moradi‐Vajargah *et al*.^66^ found the aggregated distribution of egg, larval, and adult *H. postica* in alfalfa fields. However, these mean–variance methods do not take into account the spatial location of samples.^33^ In our study, we accounted for the true spatial reference points by using SADIE or variograms to characterize the spatial distribution of *H. postica*. To our knowledge, this is the first study to use this approach to report the spatial distribution of *H. postica* and its natural enemies. Nevertheless, the results further supported the previous findings, indicating that alfalfa weevil aggregation levels varied with insect developmental stages in alfalfa fields. In contrast to the mean–variance methods reported earlier, our study did not show any indication of a shift in the type of insect distribution at different population densities.

Natural enemies can play an important role in balancing the prey density. In our study, out of the total sampling times evaluated, the spatial aggregation pattern of coccinellids was found in 57.9% and 52.6% based on variogram and SADIE, respectively. Using mean–variance methods, Evans and Trent^68^ reported similar results of spatial aggregation in *C. septempunctata*, but not in *Hippodamia convergens* (Guérin‐Méneville) and *H. quinquesignata* (Kirby) (Coleoptera: Coccinellidae). Unlike coccinellids, the aggregation pattern of *Nabis* spp. was found at a low level, 5.6% of the total datasets used for both variogram and SADIE across five fields. These results are in line with previous reports of none to moderate aggregation of *Nabis* spp. nymphal populations in soybean fields using non‐geospatial methods.^69^, ^70^, ^71^ We speculated that no aggregation of *Nabis* spp. could be due to their prey‐searching behavior, with continuous movements of individuals in the field, much more than coccinellids.

Hassell and May^72^ and Kareiva^73^ reported that the occurrence of spatial aggregation of insect predators often enhances their capacity to search and attack prey in a complex agroecosystem. Similar examples of aggregation and its role in agroecosystem based biological control have been shown in previous studies in corn^24^ and soybean^38^ fields. Despite the strong evidence of spatial aggregation of coccinellids, this study did not find a clear association not only between *H. postica* and *Nabis* spp., but also between *H. postica* and coccinellids in alfalfa fields. Several factors such as the harvest timing,^74^ the availability of food sources (main prey *versus* alternative prey), and the presence of other natural enemies can influence probability of association between coccinellids and its prey.^26^ Ghahramani *et al*.^74^ reported dissociation between aphids and coccinellid predators in alfalfa. In our study fields, the population of pea aphids (*Acyrthosiphon pisum* Harr.) (Hemiptera: Aphididae) was high, particularly during the second (JW 25) and third (JW 31) sampling periods (G. Shrestha, pers. obs.). It is known that coccinellids prefer aphids over their alternative prey that includes *H. postica*, and this could have resulted in a weak association between *H. postica* and coccinellids in these alfalfa fields. Asynchronous distribution between prey and natural enemies can be useful in certain site‐specific pest management situations such as applying insecticide when populations of prey, but not natural enemies, aggregate in the field. Under this scenario, properly timed insecticide applications would be less disruptive to natural enemy populations.^74^

The range value of the variogram models, which indicate aggregated distribution patterns (i.e. spherical, exponential and Gaussian), can be used to develop reliable sampling methods for insect pest monitoring and population assessment. Sweep net, stem‐count, and shake‐bucket are the most common sampling methods that alfalfa growers practice for scouting and monitoring of alfalfa weevil, particularly for the larval population. It is recommended to sample randomly from several locations in a field to determine the economic threshold level.^5^ However, due to the limited information available on where and how far to scout and monitor alfalfa weevil, these sampling methods are often laborious and time‐consuming, specifically in large commercial alfalfa fields.

Our study is the first to demonstrate that *H. postica* samples are spatially dependent on average distances of 22.3 m for larvae and 14.7 m for adults. The integration of range value information (specifically for larvae) into sampling methods may help to improve current alfalfa weevil larvae scouting and monitoring programs. The range value of the variogram can be used to develop sampling plans for two purposes. If the intention is to develop *H. postica* abundance hot‐spot maps and conduct site‐specific pest management as it has been used for some agricultural pests,^75^, ^76^ the sampling distance should be lower than the average aggregation distance (i.e. 22.7 m). However, for alfalfa growers, this approach may not be pragmatic as developing distribution maps requires intensive sampling points from the entire field, and also needs technical expertise for data processing and map development. If the intended use of the sampling is to take independent samples in order to determine the threshold values for insecticide treatment, the sampling distance should be higher than the average range value of the variogram.^34^, ^35^, ^58^, ^77^ This may be the most practical utility of the spatial‐distribution of a sampling plan for *H. postica* management in alfalfa fields.

In our study, coccinellid predators showed a spatial dependency within the distance of 16.6 m for most of the sampling times (~58%). The minimum sampling distance for coccinellids to obtain independent samples is 17 m, and this information can be integrated to develop a comprehensive sampling plan. Since the majority of the sampling time (> 94%) datasets indicated a random distribution of *Nabis* spp. within the field, the comprehensive sampling plan developed for *H. postica* and coccinellids should also work for *Nabis* spp. as minimum sampling distance does not apply to the randomly‐distributed population. A total of 81 sample points in each field were used to obtain an empirical minimum sampling distance using variogram analyses. With this new sampling distance guidelines, a high level of sampling intensity is not required for routine pest sampling. The sample size can be reduced by 50% to make the sampling plan more cost‐effective under field conditions. Based on the spatial distribution information generated in our study, we recommend a systematic sampling scheme, including the use of a minimum of 23 m sampling distance (based on the range value of semivariogram of weevil larvae) and a minimum of 40 sample points in a grid when conducting alfalfa weevil and predator sampling when using either sweep net or damaged stem counts. This sampling plan should provide a reasonable estimation of alfalfa weevil larvae and its natural enemies populations in the alfalfa field. This allows the alfalfa producers to accurately estimate the weevil population as well as predator(s) density within the field accurately. This approach helps to reduce the unnecessary use of pesticides by properly determining the need and timing for insecticide applications. These are important aspects of integrated pest management (IPM) in order to reduce environmental pollution, promote biological control, minimize the risks of pesticide resistance, and overall reduce the pesticide‐related expense.^24^, ^36^, ^38^

In conclusion, our study was able to characterize the spatial and temporal distribution of *H. postica* and the population of its two natural enemies (coccinellids and nabids) in irrigated alfalfa fields and develop a comprehensive sampling plan for their population assessment. Although this study was conducted in Montana, the study results and sampling recommendations should apply to other irrigated alfalfa producing regions in the western United States that include Pacific Northwest, California, Arizona, New Mexico, and others. Future studies can validate this sampling scheme for major alfalfa‐growing areas in the western United States and examine the spatial distribution of additional natural enemies, such as parasitoids, to improve pest management programs for alfalfa weevil.

## CONFLICT OF INTERESTS

The authors declare no conflict of interest. Permission to collect insect samples was granted by local private landowners of Pondera County of Montana: Jeremy Curry, Tony Verstrate, John Balkenbush and Zane Drishinski. The research activities reported here did not involve, pose a risk to, or harm any endangered or protected species.

## Endnote

1 Missing cells indicates that insect counts were insufficient to conduct aggregation analysis.
